# Wnt signaling in the regulation of adult hippocampal neurogenesis

**DOI:** 10.3389/fncel.2013.00100

**Published:** 2013-06-26

**Authors:** Lorena Varela-Nallar, Nibaldo C. Inestrosa

**Affiliations:** ^1^Centro de Investigaciones Biomédicas, Facultad de Ciencias Biológicas y Facultad de Medicina, Universidad Andrés BelloSantiago, Chile; ^2^Centro de Envejecimiento y Regeneración, Departamento de Biología Celular y Molecular, Facultad de Ciencias Biológicas, Pontificia Universidad Católica de ChileSantiago, Chile

**Keywords:** neurogenesis, hippocampus, adult hippocampal progenitor (AHP), subgranular zone (SGZ), Wnt signaling pathway, β-catenin

## Abstract

In the adult brain new neurons are continuously generated mainly in two regions, the subventricular zone (SVZ) of the lateral ventricles and the subgranular zone (SGZ) in the hippocampal dentate gyrus. In the SGZ, radial neural stem cells (NSCs) give rise to granule cells that integrate into the hippocampal circuitry and are relevant for the plasticity of the hippocampus. Loss of neurogenesis impairs learning and memory, suggesting that this process is important for adult hippocampal function. Adult neurogenesis is tightly regulated by multiple signaling pathways, including the canonical Wnt/β-catenin pathway. This pathway plays important roles during the development of neuronal circuits and in the adult brain it modulates synaptic transmission and plasticity. Here, we review current knowledge on the regulation of adult hippocampal neurogenesis by the Wnt/β-catenin signaling cascade and the potential mechanisms involved in this regulation. Also we discuss the evidence supporting that the canonical Wnt pathway is part of the signaling mechanisms involved in the regulation of neurogenesis in different physiological conditions. Finally, some unsolved questions regarding the Wnt-mediated regulation of neurogenesis are discussed.

## Introduction

**Figure 1:**
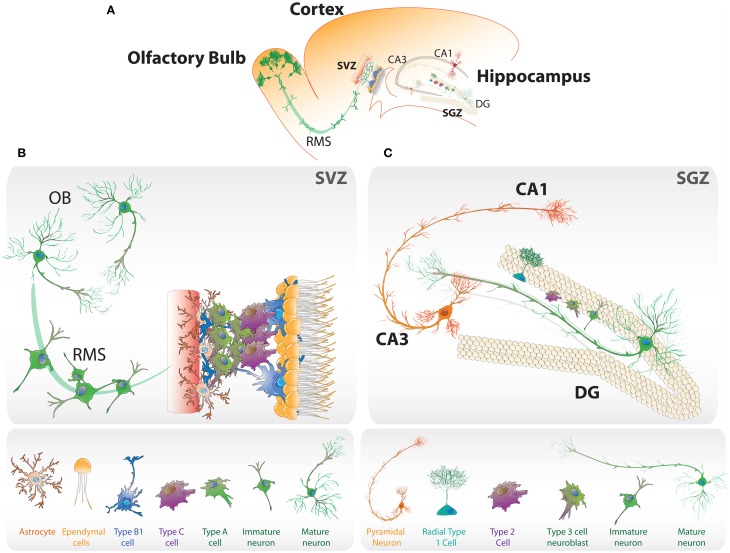
**Neurogenic niches in the adult brain. (A)** Schematic representation of the neurogenic regions in the adult rodent brain: the subgranular zone (SGZ) in dentate gyrus (DG) of the hippocampus, and the subventricular zone (SVZ) in the later wall of the lateral ventricles. **(B)** SVZ niche composed of type B1 cells, that corresponds to neural stem cells, type C cells that rapidly proliferate and type A neuroblasts, which migrate through the rostral migratory stream (RMS) to the olfactory bulb (OB) where they mature into interneurons. **(C)** Neurogenesis in the SGZ. Radial type 1 cells give rise to type 2 cells that differentiate into type 3 neuroblasts that become immature neurons and then mature into granule neurons that migrate into the granule cell layer.

The adult brain is able to continuously generate new neurons, a process known as neurogenesis, which has been reported in a number of mammalian species. Adult neurogenesis occurs mainly in two specific brain regions, the subventricular zone (SVZ) of the lateral ventricles and the subgranular zone (SGZ) in the hippocampal dentate gyrus (Alvarez-Buylla and Garcia-Verdugo, 2002; Zhao et al., 2008) (Figure 1A). Through intrinsic and extrinsic factors adult neurogenesis is tightly regulated to allow the maintenance and self-renewal of the stem cell pool and the generation of fully functional neurons. Here, we review the findings specifically supporting the role of the Wnt pathway in the adult hippocampal neurogenesis, and also discuss different studies indicating that this pathway is part of the signaling mechanisms involved in the regulation of neurogenesis by different physiological conditions.

Adult neurogenesis is a multistep process. In the SVZ astrocyte-like neural stem cells (NSCs), called type B1 cells, generate type C cells that rapidly proliferate and give rise to type A neuroblasts (Figure 1B). These cells migrate through the rostral migratory stream to the olfactory bulb where they became interneurons (Alvarez-Buylla and Garcia-Verdugo, 2002). In the SGZ, NSCs that give rise to granule cells are present at the border between the hilus and the granule cell layer (Gage, 2000). Glial fibrillary acidic protein (GFAP)-expressing radial glia-like cells, which extend a single radial process toward the molecular layer (Figure 1C), are proposed to be the stem cells or undifferentiated precursors that generate dentate granule neurons (Seri et al., 2001; Kempermann et al., 2004). These progenitors (type 1 cells) that are slowly dividing or quiescent, also express nestin and the transcription factor Sox2. A morphologically distinct class of type 1 cells that has horizontal processes has also been identified (Lugert et al., 2010). The horizontal and radial progenitors have different proliferation rate and respond differently to neurogenic stimuli (Suh et al., 2007; Lugert et al., 2010), suggesting that in the SGZ there are different populations of progenitors with different properties, increasing the complexity of the cellular and molecular mechanisms underlying regulation of adult neurogenesis. Intrinsic properties of NSCs present in the SGZ, such as self-renewing and multipotency, are still a matter of debate (Bonaguidi et al., 2011; Encinas et al., 2011; Ming and Song, 2011). These cells can be isolated and cultured generating self-renewing cells that can differentiate into neurons (Palmer et al., 1997; Peltier et al., 2010).

When activated, type 1 cells give rise to fast proliferating type 2 cells or transit-amplifying progenitors that express nestin and Sox2 but not GFAP (Kempermann et al., 2004). After limited number of cell divisions, type 2 cells commit to the neuronal lineage generating type 3 cells or neuroblasts which are proliferative and express doublecortin (DCX) but not nestin (Kronenberg et al., 2003). Neuroblasts then became immature neurons that extend dendrites toward the molecular layer and project their axons through the hilus toward the CA3 region (Figure 1C), and during several weeks mature into functional dentate granule neurons that are integrated into the pre-existing hippocampal circuitry and are located mainly in the inner granular cell layer of the dentate gyrus (Van Praag et al., 2002; Zhao et al., 2006; Mathews et al., 2010). Thus, neurogenesis can be divided into sequential stages: activation of quiescent stem cells, proliferation, neuronal fate specification, maturation and integration of newborn neurons.

For the continuous generation of new neurons in the adult brain there should be a tight regulation of the sequential steps of neurogenesis. Intrinsic factors should coordinate the proper progression of neurogenesis and preserve the stem cell pool. An impaired maintenance of the quiescent pool may result in an excessive proliferation and differentiation and ultimately in the depletion of precursor cells. If commitment and differentiation of precursor cells into neurons is disturbed, there could be no generation of new neurons even in the presence of the stem cells pool. Therefore, there should be a balance between the self-renewal and maintenance of stem cells and the differentiation into neurons. Several signaling molecules regulate hippocampal neurogenesis including Wnt, Notch, sonic hedgehog (Shh), bone morphogenetic proteins (BMP), growth and neurotrophic factors and neurotransmitters (Suh et al., 2009; Schwarz et al., 2012; Faigle and Song, 2013). Also, there is a fine tuned transcriptional control involving a number of transcriptional factors and epigenetic mechanisms that will coordinate the progression of neurogenesis (Ming and Song, 2011; Hsieh, 2012; Schwarz et al., 2012). In addition, the rate of proliferation, differentiation, and survival of newborn neurons in the hippocampus can be modulated by different physiological stimuli such as hippocampal-dependent learning and neuronal activity, exposure to environmental enrichment (EE), running and stress (Kempermann et al., 1997; Gould et al., 1998; Van Praag et al., 1999; Dobrossy et al., 2003; Drapeau et al., 2007; Piatti et al., 2011; Song et al., 2012).

Increasing evidence indicate that adult neurogenesis is important for hippocampal function, being relevant for the plasticity of the hippocampal network (Snyder et al., 2001; Mongiat and Schinder, 2011). Newborn neurons impact the hippocampal circuitry mainly while still immature, when these cells have distinct functional properties showing increased excitability and reduced GABAergic inhibition (Wang et al., 2000; Schmidt-Hieber et al., 2004; Esposito et al., 2005; Ge et al., 2007; Marin-Burgin et al., 2012). Loss of neurogenesis impairs learning and memory indicating that this process has functional implications for the adult brain (Reviewed in Deng et al., 2010; Koehl and Abrous, 2011; Marin-Burgin and Schinder, 2012).

## The Wnt/β-catenin signaling pathway in the regulation of adult hippocampal neurogenesis

Wnts compromise a large family of secreted glycoproteins that are part of the signaling molecules regulating several aspects of development such as axis formation and midbrain development (Van Amerongen and Nusse, 2009; Nusse and Varmus, 2012). The interaction of a Wnt protein with members of the Frizzled (Fz) family of seven-pass transmembrane cell-surface receptors triggers the activation of the Wnt signaling pathway (Gordon and Nusse, 2006; Wang et al., 2006; Schulte, 2010). In mammals 19 Wnt members have been found and 10 members of the Fz family have been identified. In addition, receptor-like tyrosine kinase (Ryk) and receptor tyrosine kinase-like orphan receptor (Ror2) have been identified as alternative Wnt receptors (Oishi et al., 2003; Keeble et al., 2006; Ho et al., 2012). Downstream Wnt receptors, different Wnt signaling cascades may be activated: the Wnt/β-catenin or canonical pathway that involves gene transcription, and the β-catenin-independent or non-canonical pathways that induce either an increase in intracellular calcium concentration or activation of the c-Jun-N-terminal kinase (JNK) cascade (Veeman et al., 2003; Gordon and Nusse, 2006; Angers and Moon, 2009).

In the canonical Wnt/β-catenin signaling low-density lipoprotein receptor-related proteins 5 and 6 (LRP5/6) serve as co-receptors for Wnt ligands. Activation of the canonical signaling pathway activates the protein Dishevelled (Dvl) by phosphorylation, and triggers the stabilization of cytoplasmic β-catenin, that in the absence of Wnt stimulation is ubiquitinated and constantly degraded in the proteasome (Aberle et al., 1997). β-catenin enters the nucleus and binds to members of the family of T-cell factor (TCF) and lymphoid enhancer factor (Lef) and this binding activates transcription by displacing the repression of Wnt target genes (Nusse and Varmus, 2012).

During development, Wnt signaling is fundamental for the proper development of cortex and hippocampus (Li and Pleasure, 2005; Machon et al., 2007). Wnt signaling promotes self-renewal of cortical neural progenitors and the differentiation of these progenitors in a stage specific manner (Hirabayashi et al., 2004; Munji et al., 2011). During early neurogenesis the Wnt pathway promotes self-renewal and maintains neural progenitors (Chenn and Walsh, 2002; Machon et al., 2007; Wrobel et al., 2007), while it induces the differentiation of intermediate progenitors during mid and late neurogenesis (Munji et al., 2011). In addition to the key roles of the Wnt pathway during development, it has proved to be important in the adult brain, where it regulates synapse formation, neurotransmission and plasticity, and neurogenesis (Lie et al., 2005; Adachi et al., 2007; Toledo et al., 2008; Kuwabara et al., 2009; Inestrosa and Arenas, 2010). Consistent with the previous feature, in the adult brain most of the key components of the Wnt signaling including Wnts and Fz receptors are expressed (Shimogori et al., 2004; Chen et al., 2006; Chacon et al., 2008).

Different studies have shown that the Wnt/β-catenin pathway is involved in adult hippocampal neurogenesis. In the BAT-gal reporter mouse expressing a β-catenin-activated transgene with nuclear β-galactosidase under the control of TCF/Lef (Maretto et al., 2003), it was determined that the Wnt/β-catenin pathway is active in the SGZ and the dentate granule cell layer in the adult hippocampus (Lie et al., 2005). In addition, cultured adult hippocampal progenitors (AHPs) express key components of the Wnt/β-catenin signaling pathway including some Fz receptors (Lie et al., 2005; Wexler et al., 2009). Adult hippocampal astrocytes express Wnt-3, and it was shown that Wnts derived from hippocampal astrocytes stimulate Wnt/β-catenin signaling in isolated AHPs and induce the differentiation of these progenitors into neurons, since the differentiation induced by co-culture with astrocytes was reduced in the presence of the Wnt inhibitor soluble Frizzled-related protein 2 and 3 (sFRP2/3) (Lie et al., 2005). In addition to astrocytes-derived Wnts, there is an autocrine Wnt signaling activity in AHPs (Wexler et al., 2009). Interestingly, inhibition of the autocrine Wnt stimulation increases the number of neurons formed and depletes multipotent progenitors indicating that this autocrine pathway supports the proliferation and multipotency of stem cells and therefore, it may preserve the balance between NSC maintenance and differentiation (Wexler et al., 2009). Therefore, Wnts are important for both, the maintenance of the stem cell pool and the differentiation of newborn neurons.

Regulation of adult neurogenesis by Wnt signaling was also demonstrated *in vivo* by stereotactic injection of lentiviral vectors expressing Wnt-3 or a secreted mutant Wnt-1 protein that blocks the Wnt signaling (Lie et al., 2005). Wnt signaling inhibition reduced proliferation and neurogenesis in the SGZ, while activation of the Wnt signaling increased neurogenesis (Lie et al., 2005). Later on, and by using the same lentiviral approach to block Wnt signaling activation in the dentate gyrus of adult rats, it was shown that reduction of neurogenesis by Wnt inhibition impaired long-term retention of spatial memory and object recognition memory, indicating that Wnt-mediated adult hippocampal neurogenesis contributes to hippocampal function (Jessberger et al., 2009).

The importance of the Wnt pathway in neurogenesis is also supported by studies focusing on the role of a key component of the Wnt/β-catenin pathway, the enzyme glycogen synthase kinase-3β (GSK-3β). In the absence of Wnt stimulation, β-catenin is phosphorylated by GSK-3β in a multiprotein complex composed also of the scaffold protein axin and adenomatous polyposis coli (APC) (Hart et al., 1998; Ikeda et al., 1998; Itoh et al., 1998; Kishida et al., 1998; Sakanaka et al., 1998). Phosphorylated β-catenin is recognized by β-TrCP, which is part of an E3 ubiquitin ligase complex, is ubiquitinated and subsequently degraded (Liu et al., 2002). Activation of the Wnt/β-catenin pathway inhibits the degradation pathway and induces the cytoplasmic stabilization of β-catenin and the transcription of Wnt target genes (Logan and Nusse, 2004). *In vitro* experiments show that treatment with the GSK-3β inhibitor lithium induces the proliferation of AHPs (Wexler et al., 2008). *In vivo* treatment with lithium was also shown to stimulate proliferation and neuronal fate specification in a mouse model of Alzheimer's disease (Fiorentini et al., 2010). Besides, a decreased neurogenesis was observed in a GSK-3 knock-in mouse carrying mutations to block inhibitory phosphorylation of the kinase (Eom and Jope, 2009). In this model, it was suggested that the impaired neurogenesis was not consequence of the effects of GSK-3 in neural progenitor cells but rather by alterations in the external support for the proliferation of these cells. GSK-3β has also been involved in the disrupted in schizophrenia 1 (DISC1)-mediated regulation of adult neurogenesis. DISC-1 is a schizophrenia susceptibility gene that regulates multiple steps of neurogenesis (Duan et al., 2007; Mao et al., 2009). Interestingly, DISC1 protein inhibits GSK-3β activity, and it is required for Wnt-3a-induced proliferation of cultured AHP cells and β-catenin-dependent transcription (Mao et al., 2009). The impairment in progenitor cell proliferation caused by DISC1 knockdown could be rescued by over expression of stabilized β-catenin. *In vivo*, cell proliferation in the adult dentate gyrus could be rescued by administration of a GSK-3β inhibitor, which also suppressed schizophrenia- and depression-like behaviors caused by DISC1 loss of function (Mao et al., 2009). These findings link the DISC1-mediated regulation of neurogenesis with downstream components of the Wnt/β-catenin signaling pathway.

Recently, a negative effect on neurogenesis was determined for two Wnt inhibitors, Dickkopf 1 (Dkk1) and sFRP3 (Jang et al., 2013; Seib et al., 2013). Dkk1 binds LRP co-receptors preventing the formation of the Fz/LRP complex and the consequent activation of the Wnt signaling cascade (Clevers and Nusse, 2012); sFRPs bind to Wnts preventing its interaction with cellular receptors and the activation of the Wnt pathway (Rattner et al., 1997). Inducible deletion of Dkk1 in the adult CNS resulted in an increased self-renewal of neural progenitors and increased generation of immature neurons (Seib et al., 2013). On the other hand, sFRP3 knockdown in the dentate gyrus through a lentiviral approach increased Wnt signaling and increased neural progenitor proliferation (Jang et al., 2013). Interestingly, deletion of Dkk1 and sFRP3 knockdown resulted in an increased dendrite complexity of immature neurons indicating that Wnt signaling is important for dendritic development of newborn neurons (Jang et al., 2013; Seib et al., 2013). The negative effect of both inhibitors on neurogenesis suggests that suppression of Wnt signaling by secreted factors could be a regulatory mechanism to dynamically modulate neurogenesis under physiological and pathological stimulation as we will discuss afterward.

Also, the Wnt pathway is part of the signaling mechanisms of the orphan nuclear receptor TLX (also known as NR2E1), an important regulator of NSC maintenance and self-renewal in embryonic and adult brains (Shi et al., 2004; Li et al., 2008b) that is required for adult neurogenesis in the SVZ (Liu et al., 2008) and SGZ (Zhang et al., 2008). It was determined that TLX activates the Wnt/β-catenin pathway in adult mouse NSC to stimulate proliferation and self-renewal by activating the expression of Wnt-7a through binding to the two TLX binding sites present in the Wnt-7a gene promoter (Qu et al., 2010). In accordance, Wnt-7a expression was found down-regulated in TLX-null mice. This study also revealed that Wnt-7a is important for adult NSC proliferation *in vivo*, since a decreased proliferation was observed in the SGZ and SVZ of adult Wnt-7a knockout mice. In TLX^−/−^ mice, intracranial lentiviral transduction of active β-catenin led to a considerable rescue of cell proliferation in the SVZ, suggesting that Wnt/β-catenin acts downstream of TLX to regulate NSC proliferation *in vivo* (Qu et al., 2010).

Although it is out of the scope of the present review, it is important to mention that other studies have demonstrated that neurogenesis in the SVZ is also Wnt-regulated. Retrovirus-mediated expression of a stabilized β-catenin *in vivo* promoted the proliferation of type C cells and inhibited their differentiation into neuroblasts (Adachi et al., 2007). Also in the SVZ, transduction of the β-catenin inhibitor axin by intracranial lentiviral delivery decreased cell proliferation (Qu et al., 2010), further supporting a role for Wnt/β-catenin signaling in NSC proliferation in the neurogenic areas of the adult brain.

Although the Wnt/β-catenin pathway is required for different aspects of adult hippocampal neurogenesis, excessive β-catenin signaling may impair the maturation of adult born neurons. It was determined that the impaired dendritic refinement in adult born dentate granule cells observed by primary cilia loss is a consequence of an abnormal enhancement of β-catenin signaling in newborn neurons as conditional knockout of β-catenin reversed the decrease observed in the total dendritic length in these neurons (Kumamoto et al., 2012). Moreover, expression of a constitutively active β-catenin suppresses the dendritic refinement of newborn neurons between 14 and 21 after born (Kumamoto et al., 2012). Therefore, impaired Wnt/β-catenin signaling after migration of newborn neurons may have detrimental consequences, suggesting that there should be a fine tuned activation of β-catenin signaling to allow for proper maturation and integration of newborn neurons.

## Wnt/β-catenin pathway as part of the signaling mechanisms involved in the regulation of neurogenesis in different physiological conditions

In addition to the discussed evidence directly pointing to the involvement of the Wnt signaling pathway in the proliferation and differentiation of adult neural progenitor cells, studies have indicated this pathway as part of the mechanisms involved in the regulation of neurogenesis under some physiological conditions.

### Aging

During lifespan there is a progressive reduction of hippocampal neurogenesis that have been evidenced in different species (Kuhn et al., 1996; Gould et al., 1999; Leuner et al., 2007; Olariu et al., 2007; Varela-Nallar et al., 2010b), including humans (Knoth et al., 2010). The generation of new neurons in the adult human dentate gyrus was originally evidenced in postmortem tissues from patients who were treated with the thymidine analog bromodeoxyuridine (BrdU) (Eriksson et al., 1998). Thereafter, several studies have suggested that neurogenesis in humans can be regulated by different physiological and pathological conditions (Jin et al., 2004; Li et al., 2008a; Gerber et al., 2009; Mattiesen et al., 2009; Boldrini et al., 2012). As determined in other species, it was shown a reduction of DCX expressing cells with increasing age suggesting that there is an age-related decline in the generation of new neurons in the human hippocampus (Knoth et al., 2010).

In rodents, evidence indicate that a decline in Wnt signaling is associated to the age-dependent reduction in neurogenesis. During aging, the levels of Wnt-3 protein in hippocampal astrocytes and also the number of Wnt-3-secreting astrocytes decline (Okamoto et al., 2011). This finding is important since as previously mentioned, Wnts derived from hippocampal astrocytes stimulate Wnt/β-catenin signaling in neural progenitors and induce its neural differentiation (Lie et al., 2005). It was shown in rats that there is a progressive decrease in the expression of Wnt-3 and Wnt-3a in the dentate gyrus between 2 and 22 month, concomitantly with a decrease in the expression of NeuroD1 (Okamoto et al., 2011). NeuroD1 is a basic helix-loop-helix transcription factor important for the generation of granule cells and olfactory neurons in the embryonic and adult brain (Gao et al., 2009). This suggests that the decline in Wnt-3/Wnt-3a expression in astrocytes may cause the decreased expression of proneural genes and in consequence the decrease in neurogenesis. More recently, the Wnt inhibitor Dkk1 was also involved in the age-related decline in neurogenesis (Seib et al., 2013). As mentioned, Dkk1 is a suppressor of NSCs proliferation. The expression of Dkk1 increases with age, suggesting that suppression of Wnt signaling by this inhibitor may downregulate neurogenesis during aging (Seib et al., 2013). Whether Wnt is associated to the age-related decline in neurogenesis in humans is not known, but it was reported an association between Wnt-3 levels and cell proliferation in the human hippocampus (Gerber et al., 2009), suggesting that this pathway may also regulate neurogenesis in the human brain.

### Exercise

Running, which is one of the physiological stimuli that strongly stimulates adult neurogenesis in the SGZ (Van Praag et al., 1999), modulates the expression of genes involved in Wnt signaling (Stranahan et al., 2010). Moreover, running was found to significantly increase the expression of Wnt-3 in astrocytes of the dentate gyrus (Okamoto et al., 2011) and to increase the population of Wnt-3 expressing cells in young and aged mice. More recently, it was shown that exercise also regulates the expression of the Wnt inhibitor sFRP3, which is a suppressor of adult hippocampal neurogenesis (Jang et al., 2013). Exercise as well as electroconvulsive stimulation (ECS) decreased the expression of sFRP3 in dentate granule neurons, and infusion of sFRP3 into the dentate gyrus abolished the ECS-induced increase of neural progenitor proliferation (Jang et al., 2013), suggesting that the reduction of sFRP3 levels is important for the activity-mediated increase in neurogenesis. In addition to the reported increase of Wnt-3 levels by running, this finding strongly implicates the Wnt pathway as a signaling mechanisms involved in the excercise-mediated increase in neurogenesis.

### Hypoxia

An association between hypoxia and neurogenesis in embryonic and adult brain has been demonstrated by different studies. Increased neurogenesis in the rodent dentate gyrus was observed in response to global ischemia (Liu et al., 1998). Also, intermittent hypobaric hypoxia regimen promoted the proliferation of endogenous neural progenitors leading to more newborn neurons in the hippocampus of adult rats (Zhu et al., 2010). Interestingly, there has been suggested an association between hypoxia and the Wnt/β-catenin pathway in embryonic stem cells (ESCs) and NSCs. Hypoxia increases β-catenin signaling in ESCs and increases the expression of Lef1 and TCF1 genes (Mazumdar et al., 2010). The hypoxia-mediated activation of the Wnt pathway is mediated by hypoxia-inducible transcription factor-1α (HIF-1α) that directly binds to the promoter of the Lef1 and TCF1 genes in cultured ESC under hypoxic conditions (Mazumdar et al., 2010). Moreover, it was determined in the BAT-gal reporter mouse that the Wnt/β-catenin signaling is active in low oxygen regions in the adult brain, including in the SGZ. This suggests an association between low oxygen and β-catenin signaling *in vivo*, which was shown to be dependent of HIF-1α (Mazumdar et al., 2010).

## Potential mechanisms involved in the Wnt-mediated regulation of adult hippocampal neurogenesis

How the Wnt/β-catenin signaling could regulate neurogenesis? The molecular mechanism may involve the transcriptional activation of NeuroD1 which depends on the Wnt/β-catenin signaling activation (Kuwabara et al., 2009). NeuroD1 gene promoter has overlapping DNA-binding site for Sox2 and TCF/Lef, then the activation of this gene implies activation of the canonical Wnt pathway and removal of Sox2 repression from the NeuroD1 gene promoter (Kuwabara et al., 2009). Prox1 is also a Wnt target gene that could be relevant for the neurogenic effect of the Wnt/β-catenin pathway (Karalay et al., 2011). Prox1 is expressed in newborn and mature granule cells and is required for the proper differentiation and survival of newborn granule cells, but not for the maintenance of granule cells after they have fully matured (Karalay et al., 2011). Interestingly, the promoter region of long interspersed element-1 (L1) retrotransposons, which was found to be actively retrotransposed during neurogenesis (Muotri et al., 2005; Coufal et al., 2009), contains dual binding sites for Sox2 and TCF/Lef (Kuwabara et al., 2009). Therefore, Wnt signaling activation could upregulate the expression of genes adjacent to the L1 loci that may be relevant for neurogenesis such as DCX (Okamoto et al., 2011).

In addition, the Wnt signaling pathway could directly or indirectly modulate neurogenesis through the regulation of glutamatergic neurotransmission in the hippocampus, since neural progenitor cells respond to neuronal activity as part of their differentiation program (Deisseroth et al., 2004). We and others have determined that Wnt ligands regulate synaptic assembly as well as synaptic plasticity and neurotransmission in the hippocampus. In cultured hippocampal neurons, Wnt-3a, Wnt-7a and Wnt-7b regulate pre-synaptic assembly increasing the number of pre-synaptic puncta (Ahmad-Annuar et al., 2006; Cerpa et al., 2008; Davis et al., 2008). In addition, Wnt-7a stimulates the clustering of the pre-synaptic receptor α7- nicotinic acetylcholine receptor (Farias et al., 2007), indicating that the Wnt signaling regulates the clustering of pre-synaptic receptors. Evidence indicate that these ligands are able to modulate pre-synaptic differentiation by activation of the Wnt/β-catenin signaling pathway. In accordance with the structural data, electrophysiological recordings have revealed that Wnts have modulatory effects on glutamatergic neurotransmission (Ahmad-Annuar et al., 2006; Cerpa et al., 2008; Varela-Nallar et al., 2010a; Avila et al., 2010). On adult rat hippocampal slices, Wnt-7a increases neurotransmitter release in CA3-CA1 increasing the frequency of miniature excitatory post-synaptic currents (mEPSC) (Cerpa et al., 2008). Wnt-3a is also able to increase the frequency of mEPSC in cultured hippocampal neurons (Avila et al., 2010). The synaptic effects of Wnts could regulate the generation and maturation of newborn neurons.

Importantly, the release and expression of Wnt ligands is modulated by neuronal activity (Chen et al., 2006; Wayman et al., 2006; Tabatadze et al., 2012), and incubation of adult hippocampal slices with secreted Wnt inhibitors affects glutamatergic neurotransmission (Chen et al., 2006; Varela-Nallar et al., 2010a; Cerpa et al., 2011), strongly suggesting that endogenous Wnt signaling in the brain modulates hippocampal function and could modulate the generation of new neurons.

The *in vivo* relevance of the Wnt signaling in the hippocampal function is demonstrated by the effects of EE, which increases Wnt-7a/b levels in CA3 pyramidal neurons in parallel to increasing the complexity and number of large mossy fiber terminals in the CA3 region (Gogolla et al., 2009). Interestingly, inhibiting Wnt signaling through local application of the Wnt inhibitor sFRP-1 suppressed EE effects. Also, it was determined that training in the hidden platform Morris water maze task increases the levels of Wnt-7a/b in granule cells of rat dentate gyrus but not in CA3 pyramidal cells (Tabatadze et al., 2012). The increase of hippocampal Wnt-7a/b levels was still observed 30 days after training, indicating that this is a long-lasting effect that could be associated to long-term spatial memory (Tabatadze et al., 2012). Therefore, Wnt signaling pathway is regulated by neuronal activity and regulates neurotransmission. The activity-mediated Wnt signaling activation could modulate adult hippocampal neurogenesis which may contribute to Wnt-mediated increase in hippocampal plasticity.

## Unsolved questions and future perspectives

Although the evidences indicate that Wnts are part of the signaling molecules that regulate neurogenesis in physiological conditions, there are still unsolved questions remaining. In this part we will discuss two attractive issues that from our point of view should be addressed in the future.

### Putative role of the non-canonical Wnt signaling cascades in adult hippocampal neurogenesis

One aspect that should be addressed is the potential role of non-canonical Wnt signaling cascades. There are at least two β-catenin-independent pathways: the planar cell polarity (PCP) pathway and the Ca^2+^ pathway. The PCP pathway, also known as the Wnt/JNK pathway, was originally identified in *Drosophila* where it regulates tissue polarity and cell migration (Adler, 2002; Veeman et al., 2003). This signaling pathway activates small GTPases including Rho and Rac and the protein kinase JNK, and affects cytoskeleton dynamics. It would be interesting to study whether this pathway regulates the proper polarization and migration of newborn neurons in the adult brain as it does during development. On the other hand, the activation of the Wnt/Ca^2+^ pathway triggers the increase in intracellular Ca^2+^ levels and activates the protein kinases CamKII and protein kinase C (PKC) (Veeman et al., 2003; Kohn and Moon, 2005).

We have determined that non-canonical Wnt pathways have relevant roles in the adult hippocampus. In cultured hippocampal neurons, the Wnt-5a ligand able to activate non-canonical Wnts cascades (Farias et al., 2009; Cuitino et al., 2010), plays relevant roles in synaptic structure and function. Wnt-5a increases dendritic spine morphogenesis (Varela-Nallar et al., 2010a), effect also described for Wnt-7a which increase the density and maturity of dendritic spines through a non-canonical CamKII-dependent mechanism (Ciani et al., 2011). In addition, Wnt-5a increases the clustering of the post-synaptic density protein-95 (PSD-95) (Farias et al., 2009). PSD-95 is a scaffold protein of the post-synaptic density, a multiprotein complex containing key molecules involved in the regulation of glutamate receptor targeting and trafficking and regulatory proteins relevant for neurotransmission (Li and Sheng, 2003; Han and Kim, 2008). Electrophysiological data supports the synaptic roles of Wnt-5a. Acute application of Wnt-5a increases the amplitude of field excitatory post-synaptic potentials (fEPSP) in hippocampal slices (Varela-Nallar et al., 2010a) and upregulates synaptic NMDA receptor currents facilitating induction of long-term potentiation (LTP) (Cerpa et al., 2011). Non-canonical ligands could indirectly influence the proliferation and differentiation of progenitor cells through modulating hippocampal neurotransmission, or could directly regulate synaptogenesis and connectivity of newborn neurons.

Wnt-5a also induces the recycling of functional GABA_A_ receptors on hippocampal neurons through activation of CaMKII, and modulates inhibitory synapses (Cuitino et al., 2010). GABA is critical for the proper development and maturation of adult-born neurons (Tozuka et al., 2005; Ge et al., 2006; Jagasia et al., 2009), therefore the effect of this ligand on the inhibitory synapse may influence the development of newborn neurons.

Interestingly, it was determined that training in the hidden platform Morris water maze task increased hippocampal Wnt-5a levels (Tabatadze et al., 2012), suggesting that this ligand is also regulated by activity and may regulate neurogenesis under certain stimuli.

The effect of Wnt-5a was addressed in postnatal SVZ (Pino et al., 2011). In neural precursor cells cultured from the SVZ of mice at postnatal day 5, Wnt-5a treatment increased neurite outgrowth, effect completely different to Wnt-3a treatment that inhibited neurite development (Pino et al., 2011), suggesting that non-canonical pathway increases neurite complexity while canonical pathway suppresses dendrite maturation. Whether the same regulation takes place in the SGZ is not known.

### Wnt receptor context could specifically regulate the different stages of neurogenesis

Considering that different populations of stem and progenitor cells have been identified in the SGZ, with different proliferation rates and response to neurogenic stimuli, it should be interesting to evaluate whether these populations have different ability to respond to the signaling molecules present in the local microenvironment. Progenitor cells, as well as newborn neurons at different stages of maturity, may have different subsets of receptors and/or co-receptors for the Wnt signaling pathway, which may mimic what is observed in hippocampal neurons (Varela-Nallar et al., 2012). The expression pattern of Fz receptors during postnatal development is very different, being some of them highly expressed in adulthood and others during early postnatal development. In cultured hippocampal neurons, the distribution of Fzs is also very specific being some of them located in synaptic regions and others in the soma or growth cones of young neurons (Varela-Nallar et al., 2012). Interestingly, the distribution of Fz receptors seems to be associated to specific functions. Fz1 that is located in the synaptic region co-localizing with pre-synaptic proteins and with active synaptic vesicle recycling sites (Varela-Nallar et al., 2009), regulates pre-synaptic differentiation. Overexpression of Fz1 receptor increased the clustering of the active zone protein Bassoon (Varela-Nallar et al., 2009), involved in the structural organization of neurotransmitter release sites that is recruited early during synapse formation (Zhai et al., 2000). As well, Fz5 which is also present in synaptosomes and co-localizes with synaptic markers, modulates the synaptogenic effect of Wnt-7a (Sahores et al., 2010). Changes in the expression of this receptor modulates the density of synaptic sites in mature neurons (Sahores et al., 2010).

**Figure 2:**
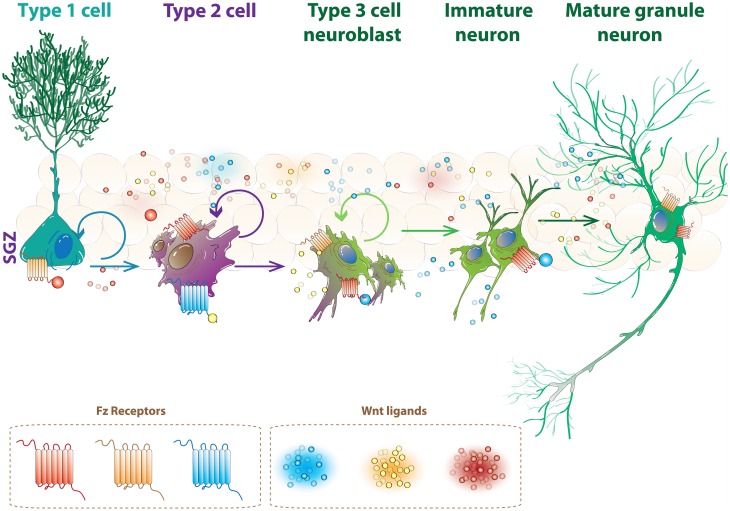
**Specific Wnt receptors could regulate the progression of neurogenesis in the hippocampus**. Schematic illustration of neurogenesis in the SGZ. As represented, stem cells, progenitor cells, immature neuron and mature granule neurons are constantly present in the dentate gyrus. Wnt ligands constitutively expressed in the hippocampus could induce different signaling cascades in these cells and regulate different stages of neurogenesis by interacting with specific receptors (see text for details).

The effects of Wnts on the proliferation of progenitor cells and neuronal differentiation could be mediated by selective Wnt/receptor complexes. As mentioned, there are 19 Wnt members, 10 Fz receptors and alternative receptors and co-receptors described in mammals, therefore there is a complex scenario in which there is a wide range of possible interactions that may specifically regulate all aspects of neurogenesis. In this context, Wnt ligands constitutively expressed in the hippocampus may induce different signaling cascades in stem cells, progenitors and newborn neurons by interacting with specific sets of receptors and co-receptors, and in that way may regulate the sequential events of neurogenesis (Figure 2).

The receptor context may also be essential for specific effects of canonical and non-canonical Wnt signaling cascades. As mentioned, Wnt-5a and Wnt-3a have differential effects in neural precursor cells cultured from postnatal SVZ (Pino et al., 2011). In this neurogenic region, the dual regulation by Wnt ligands may be achieved by region-specific expression of Wnt ligands during the specification and maturation of olfactory bulb interneurons in the SVZ, rostral migratory stream and olfactory bulb. Whether the same regulation occurs during neurogenesis in the adult SGZ it is not known. In the hippocampus, all steps of neurogenesis occur at the dentate gyrus, where there are stem cells, progenitors and newborn neurons at different maturation stages in close proximity. Therefore, factors regulating each aspect of neurogenesis should be present at the dentate gyrus. It has been proposed that radial stem cells at the hippocampus have three domains that span three different anatomical layers (Fuentealba et al., 2012), which could allow specific microenvironments during neurogenesis; however, it is also plausible that signal integration of the stage-specific cues present in the niche may be given by specific Fz receptors as we have discussed.

## Conclusions

Altogether, the reviewed evidences indicate that the Wnt pathway is relevant for the development of new neurons in the adult hippocampus. The canonical Wnt/β-catenin signaling pathway is important for the maintenance and self-renewal of the stem cell pool, and for progenitor cell proliferation, fate commitment and differentiation. Therefore, Wnt ligands are part of the signaling molecules in the SGZ that could regulate the progression of neurogenesis. Several Wnt ligands are constantly present in the adult hippocampus, but Wnt activity is also dynamically regulated by the expression and release of Wnt ligands and soluble Wnt inhibitors. This dynamic regulation of Wnt activity could be relevant for the regulation of neurogenesis under different physiological conditions.

### Conflict of interest statement

The authors declare that the research was conducted in the absence of any commercial or financial relationships that could be construed as a potential conflict of interest.
